# Distributed Multisensor Data Fusion under Unknown Correlation and Data Inconsistency

**DOI:** 10.3390/s17112472

**Published:** 2017-10-27

**Authors:** Muhammad Abu Bakr, Sukhan Lee

**Affiliations:** Intelligent Systems Research Institute, Sungkyunkwan University, Suwon, Gyeonggi-do 440-746, Korea; abubakr@skku.edu

**Keywords:** multisensor data fusion, decentralized estimation, distributed fusion, inconsistent estimates, spurious data, unknown correlation

## Abstract

The paradigm of multisensor data fusion has been evolved from a centralized architecture to a decentralized or distributed architecture along with the advancement in sensor and communication technologies. These days, distributed state estimation and data fusion has been widely explored in diverse fields of engineering and control due to its superior performance over the centralized one in terms of flexibility, robustness to failure and cost effectiveness in infrastructure and communication. However, distributed multisensor data fusion is not without technical challenges to overcome: namely, dealing with cross-correlation and inconsistency among state estimates and sensor data. In this paper, we review the key theories and methodologies of distributed multisensor data fusion available to date with a specific focus on handling unknown correlation and data inconsistency. We aim at providing readers with a unifying view out of individual theories and methodologies by presenting a formal analysis of their implications. Finally, several directions of future research are highlighted.

## 1. Introduction

Multisensor data fusion refers to the process of utilizing additional and complementary data from multiple sources to achieve inferences that are not feasible/possible from an individual data source operating independently. More specifically, multisensor data fusion is to obtain a more meaningful and precise estimate of a state by combining data from multiple sensors and model-based predictions. These days, multisensor data fusion has been widely adopted in diverse fields of application including manufacturing and process control, autonomous navigation (SLAM) [1,2], robotics, remote sensing [3], medical diagnosis, image processing and visual recognition [4,5,6,7], fault-tolerant control [8] etc., beyond traditional application domain in the military field [9]. 

The architecture of multisensor data fusion can be broadly categorized into two, depending on the way raw data are processed: (1) *Centralized fusion architecture* [10], where raw data from multiple sources is sent directly to and fused in the central node for state estimation and (2) *Distributed fusion architecture* [10,11,12], where data measured at multiple sources is processed independently at individual nodes to obtain local estimates before they are sent to the central node for fusion. Although centralized fusion can yield theoretically optimal solutions, it is not scalable to the number of nodes, i.e., processing all sensor measurements at a single location is either ineffective or infeasible as the number of nodes increases due to communication overhead and reliability degradation. The distributed fusion, on the other hand, is robust to failures and has the advantage of lower infrastructure and communication costs. 

However, distributed fusion needs to take the correlations among local estimates into consideration. This is due to the fact that local estimates can be dependent due to double counting, i.e., sharing prior information or data sources [9,13] and that distributed sensors observe data with definite physical relationships existing among their observations [14,15]. In a centralized architecture where the assumption of statistical independence is applicable, the Kalman filter (KF) [16] provides an optimal estimate in the sense of minimum mean square error (MMSE). On the other hand, in a distributed architecture where the assumption of statistical independence is not applicable, filtering without taking the cross-correlation into account may lead to divergence due to the inconsistency in fused mean and covariance [9]. In the case of known cross-correlations among data sources, the Bar-Shalom Campo (BC) formula [17,18] provides consistent fusion results for a pair of data sources. A generalization to more than two data sources with known cross-correlations is given in References [19,20,21,22].

However, it is difficult to estimate the cross-correlation among the data sources, especially with a distributed fusion architecture. For a large distributed sensor network [23], even taking account of all cross-correlations may be too expensive to carry out for fusion. Unfortunately, simply neglecting the cross-correlations results in a conservative fused mean and covariance [24]. Various methods have been proposed to cope with the problem of fusion under unknown correlations in a distributed architecture. Depending on the way that unknown cross-correlations are handled, these methods can be categorized into three groups, including (1) Data Decorrelation, where the input data sources are decorrelated before fusion based on the measurements reconstruction [25,26] or explicit elimination of double counting [27,28]; (2) Modeling Correlation, where fused solutions are obtained based on some knowledge and modeling of the unknown correlation [29,30,31,32]; and (3) Ellipsoidal Methods (EM), under the assumption of bounded cross-correlation, these methods attempt to provide a suboptimal but consistent fused solution by approximating the intersection among individual data sources without any knowledge of cross-correlation. The EM include, Covariance Intersection Method (CI) and its derivatives [33,34,35], Largest Ellipsoid Method (LE) [36], Internal Ellipsoidal Approximation (IEA) [37,38] and Ellipsoidal Intersection Method (EI) [39]. 

Another issue in sensor fusion is that sensors frequently provide spurious measurements that are difficult to predict and model. Fusion methodologies assume that the sensor measurements are affected by Gaussian noise only, and thus the covariance of the estimate provides a good approximation of all disturbances affecting the sensor measurements. However, sensors may produce inconsistent and spurious data due to unmodeled faults, including permanent sensor failures, sensor glitches, short duration spike faults, slowly developing failures due to sensor elements, etc. [40,41,42]. Fusion of inconsistent sensor data with correct data can lead to severely inaccurate results [43]. For example, when exposed to abnormalities and outliers Kalman filter would easily diverge [44]. Hence, a data validation scheme is required to identify and eliminate the inconsistencies/faults/outliers before fusion in a distributed fusion architecture. 

Multisensor data fusion in the presence of inconsistent and spurious sensor data can be broadly classified into the following three categories: (1) Model based approaches, where inconsistencies are identified and excluded based on a comparison of sensor data against a reference, obtained through a mathematical model of the system [45,46]; (2) Redundancy based approaches, where multiple sensors provide an estimate of a quantity of interest and then identify and remove the inconsistent estimates by consistency checking and majority voting [40,47]; and (3) Fusion based approaches, where the fuse covariance is enlarged to cover all local means and covariances in such a way that the fused estimate is consistent under spurious data [48,49].

This paper systematically reviews the key theories and methodologies of distributed multisensor data fusion with a specific focus on fusion under unknown correlation and fusion in the presence of inconsistent and spurious sensor data. While several general reviews of data fusion [9,50,51,52] and data inconsistency [46,53,54] exists; this paper is intended to provide readers with the methodology of fusion under unknown correlation and data inconsistency in the context of distributed data fusion. The rest of the paper is organized as follows. In Section 2 centralized and distributed fusion architectures are explained along with the causes of correlation in distributed sensor systems. Section 3 provides an overview of the Kalman filter framework and fusion in the case of known cross-correlation. In Section 4, various methods for fusion under the assumption of unknown correlation are analyzed. In Section 5, fusion of spurious and inconsistent sensor data is reviewed. Finally, the paper is concluded and several future directions of research in distributed data fusion are highlighted.

***Preliminaries:***  R and $R_{+}$ respectively define the set of real and non-negative real numbers. We denote $A\in R^{n\times m}$ as a matrix with n rows and m columns. Similarly, I denotes an identity matrix. The inverse and transpose of matrix A are denoted as $A^{-1}$ and $A^{T}$ respectively. Given positive semidefinite matrices $A,\;B\in R^{n\times n}$, that is, A, B≥0, then A≥B means A−B≥0 (A−B is positive semidefinite). Let $\hat{x}=E\left[x\right]$ and $P=E\left[xx^{T}\right]-E\left[x\right]E\left[x^{T}\right]$ denote the mean and covariance of the random vector $x\in R^{n}$ respectively. Where the notation E [*] denotes the expectation. The cross-covariance between two random vectors $x_{1},x_{2}\in R^{n}$ is represented as $P_{12}=E\left[x_{1}x_{2}{}^{T}\right]-E\left[x_{1}]E[x_{2}{}^{T}\right]$. Furthermore, due to positive semi definiteness of the covariance matrix, $P_{12}=P_{21}$. We denote the Gaussian distribution as $x\sim N\left(\hat{x},P\right)$, with mean $\hat{x}$ and covariance P. Furthermore, the Gaussian distribution $N\left(\hat{x},P\right)$, can be represented by an ellipsoid $\varepsilon \left(\hat{x},P\right)$, as $\varepsilon \left(\hat{x},P\right)=\{x\;\epsilon \;{\mathbb{R}}^{n}|\;{\left(x-\hat{x}\right)}^{T}P^{-1}\left(x-\hat{x}\right)\leq 1\}$.

## 2. Fusion Architectures

In a data fusion framework, multiple sensors provide additional and complementary data to a fusion center, where the data is combined to obtain a precise and more meaningful information about the underlying states of an object. Based on the availability and processing of raw data, the fusion architectures can be divided into Centralized and Distributed fusion architectures.

### 2.1. Centralized Fusion Architecture

**Figure 1:**
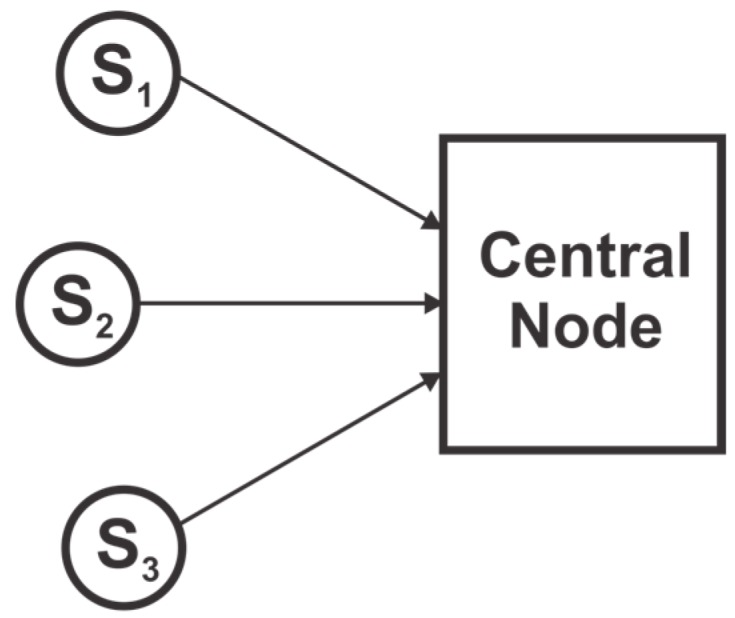
Centralized fusion architecture.

In a Centralized fusion architecture, raw data from multiple sensors is directly sent to a central fusion node, which computes state estimates and makes decisions as shown in Figure 1. Although, local sensors may pre-process the data before transmitting it to the central node, the term ‘raw data’ signify sensor measurements or pre-processed data without filtering or local fusion. Each sensor observes and provides measurements to the central system where data is filtered and fused. If the data is correctly aligned and associated, and there is no constraint on the communication bandwidth, then the centralized fusion architecture yields a theoretical optimal solution to state estimation. However, processing all the information at a central node poses various issues, such as a large computational load on the central node, large communication bandwidth requirement, the possibility of failure (due to failure of the central node) and inflexibility to changes in architecture [50,52,55].

### 2.2. Distributed Fusion Architecture

**Figure 2:**
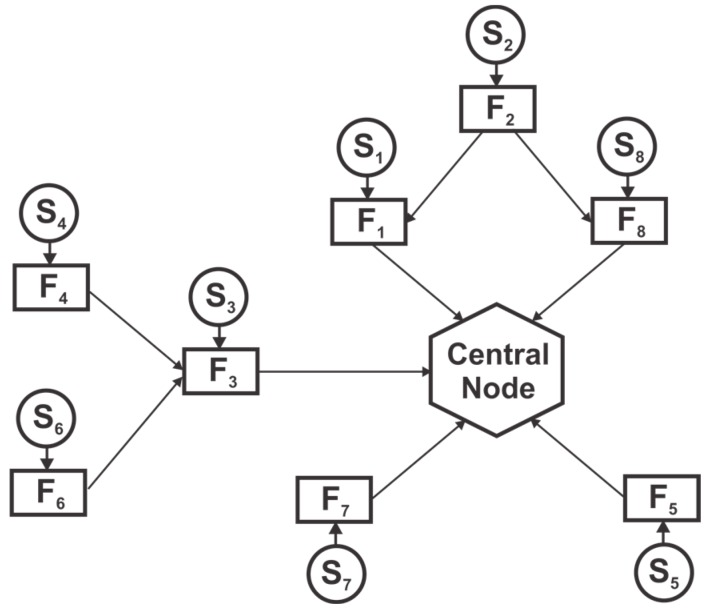
Distributed fusion architecture. Each node consists of sensor and fusion node.

Advances in sensor and communication technologies mean that each sensor node can independently process its sensor data to compute local state estimates. In most applications, the raw information is used to compute the state estimates of some quantity of interest in the form of the mean and covariance. These estimates are then communicated among sensor nodes and to the central node to form a global state estimate as depicted in Figure 2. Compared to a centralized architecture, a distributed network of sensors is superior in many settings, that is, an outstanding potential to solve the problems in a cooperative fashion, coverage of large area, and considerable increase in spatial resolution to name a few [12,52,55,56]. Furthermore, local processing of the data means a low processing load on each node due to the distribution of load, lower communication cost, flexibility to changes and robustness to failure. Still, another fusion architecture is the Decentralized one where nodes operate independently, share information with each other without any central fusion node [14,55]. Different from a distributed architecture, the decentralized architecture lacks any central node, rather each node computes the underlying system states and communicates with each other. The reason for dependencies in decentralized and distributed architectures are the same. Thus, these two architectures are categorized as one in this paper.

In general, a decentralized or distributed network of sensors cannot achieve the estimation quality of a centralized system but is inherently more flexible and robust to failure. The local sensor estimates in a distributed architecture may be correlated because observations from distributed sensors can be affected by the same process noise [15] and local estimates can be dependent due to double counting [9,13]. A distributed fusion algorithm should take into account the cross-correlation to ensure optimality and consistency. In some situations, sensor measurements may also be affected by unexpected uncertainties, that is, spike faults, permanent failure or slowly developing failure [40,49,52]. Thus, the estimates provided by sensors may be spurious and inconsistent. Hence, a data validation scheme is required to identify and eliminate inconsistent sensor estimates before the fusion process.

### 2.3. Causes of Correlation

**Figure 3:**
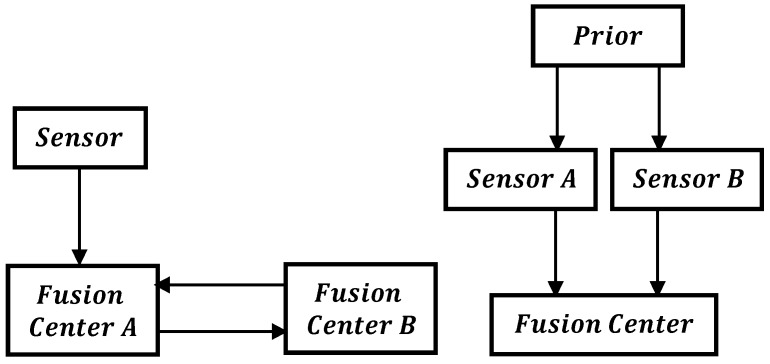
Causes of Double Counting.

A common reason for the dependencies of local estimates in a distributed sensor network is the data incest/rumor propagation/double counting of the data [9,13]. Double counting is a situation in which data is unknowingly used multiple times. This may be caused by either recirculation of the information through cyclic paths or the same information taking several paths from another sensor to the fusion node [9,55], as depicted in Figure 3. For instance, two sensor nodes A and B that are initialized with the same prior estimate ${\hat{x}}_{P}$ on the sates, i.e., ${\hat{x}}_{A}={\hat{x}}_{P}$ and ${\hat{x}}_{B}={\hat{x}}_{P}$ have correlated errors, i.e., $E\left[\left({\hat{x}}_{A}-x\right)\left({\hat{x}}_{B}-x\right)\right]=P_{A}=P_{B}=P_{P}.$ The separation of common sensor data from independent data become more difficult as the data is further processed along the communication paths and network topology [55], and the source of the common data become unknown. Fusing the local sensor estimates without accounting for the common information results in an underestimated error covariance. Another reason for interdependencies is the common process noise [14,35]. A typical example of this is the decentralized monitoring system for chemical processes [14]. The temperature measured from the pressure information combined with a reaction model and the temperature measured directly from the temperature nodes are dependent. Similarly, a KF estimating position and another KF maintaining the orientation of a vehicle using the same sensor information results in a dependent position and orientation error [14].

## 3. Distributed Data Fusion

This section focuses on various data fusion algorithms. First, a Kalman filter and its variants are overviewed, and this is followed by fusion of multiple data sources under exactly known cross-covariance.

### 3.1. Kalman Filter

Kalman filter (KF) [16] is a fundamental tool that can be used to analyze and solve a broad class of estimation problems. It has been extensively used for various purposes, including estimation, tracking, sensor fusion etc. The KF framework consists of a prediction based on the system matrix of the underlying state vectors, followed by an update provided by sensor measurements. Consider a linear dynamic system with the following system model and measurement equation,

(1) $${\hat{x}}_{k}=A_{k}{\hat{x}}_{k-1}+B_{k}u_{k-1}+w_{k}$$

(2) $$z_{k}=H_{k}{\hat{x}}_{k}+v_{k}$$

where k represents the discrete-time index, $A_{k}$ is the system matrix, $B_{k}$ the input matrix, $u_{k-1}$ the input vector, and ${\hat{x}}_{k-1}$ the process states. The process noise $w_{k}$ and measurement noise $v_{k}$ are white, zero mean, uncorrelated Gaussian with covariance $Q_{k}$ and $R_{k}$ respectively. The Kalman filter prediction of the state estimate and its error covariance is given as [57],

(3) $$\hat{x}{}_{k}^{-}=A_{k}{\hat{x}}_{k-1}+B_{k}u_{k-1}$$

(4) $$P_{k}^{-}=A_{k}P_{k-1}A_{k}^{T}+Q_{k}$$

**Figure 4:**
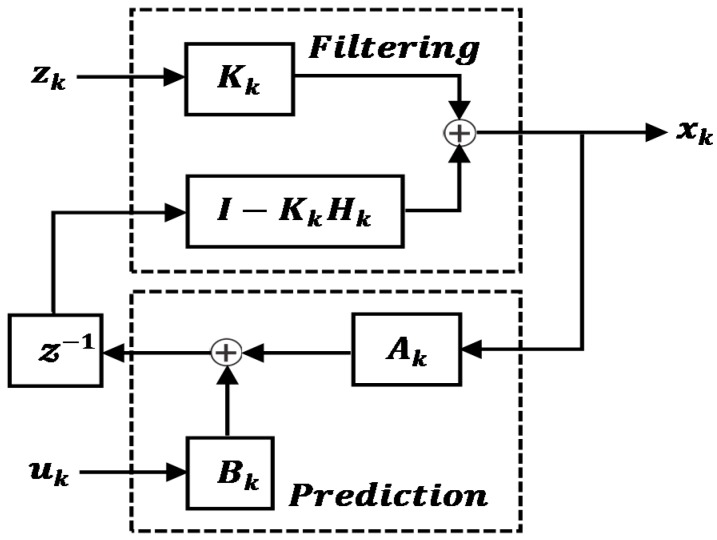
Kalman filter Prediction-Update procedure ($z^{-1}$ represents a unit time delay).

The predicted estimate $\hat{x}{}_{k}^{-}$ and error covariance $P_{k}^{-}$ are then combined with the received sensor measurement $z_{k}$ with covariance $R_{k}$ to obtain an updated estimate and error covariance matrix,

(5) $${\hat{x}}_{k}=\hat{x}{}_{k}^{-}+K_{k}\left(z_{k}-H_{k}\hat{x}{}_{k}^{-}\right)$$

(6) $$P_{k}=\left(I-K_{k}H_{k}\right)P_{k}^{-}{\left(I-K_{k}H_{k}\right)}^{T}+K_{k}R_{k}K_{k}^{T}$$

where $K_{k}$ is the Kalman gain and calculated as, $K_{k}=P_{k}^{-}H_{k}^{T}(H_{k}P_{k}^{-}H_{k}^{T}+R_{k}{)}^{-1}$. Figure 4 depicts the prediction and update cycle of the KF. The KF has been further modified as an Extended Kalman Filter (EKF) [58] and Unscented Kalman Filter (UKF) [59,60] to address the issue of non-linearity in the state estimation. The EKF and UKF are often employed in the field of robotics for tracking and navigation. In References [61,62], an information theoretic approach to KF has been proposed. The Information filter (IF) is a KF that estimates the information state vector, y, defined as $y=P^{-1}x$, where x is the state vector and P its covariance. The inverse covariance matrix $P^{-1}$ is equal to the Fisher information matrix and maximizing the Fisher information about the state is related to MMSE estimation. The representation of KF as an IF is beneficial when the state vector is larger than the measurement vector [24,62]. Furthermore, a KF implementation for the update stage become very complex when the cross-correlation between observation innovations are accounted for. The simple additive nature of the update stage makes the IF highly attractive for multisensor estimation [63].

### 3.2. Fusion under Known Correlation

One simplification in distributed estimation is the assumption of conditional independence of estimates. However, ignoring the cross-correlation in a distributed architecture leads to inconsistent results, which can result in a divergence of fusion algorithm [9,24]. Various methods have been devised to incorporate known cross-correlation for state estimation and fusion. A well-known result is the Bar-Shalom Campo (BC) formula [17], which is given as,

(7) $$P_{f}=P_{1}-\left(P_{1}-P_{12}\right){\left(P_{1}+P_{2}-P_{12}-P_{21}\right)}^{-1}\left(P_{1}-P_{21}\right)$$

(8) $${\hat{x}}_{f}=\left(P_{2}-P_{21}\right){\left(P_{1}+P_{2}-P_{12}-P_{21}\right)}^{-1}{\hat{x}}_{1}+\left(P_{1}-P_{12}\right){\left(P_{1}+P_{2}-P_{12}-P_{21}\right)}^{-1}{\hat{x}}_{2}$$

The BC formula provides a consistent fusion result in the sense of Maximum Likelihood [18] for a pair of redundant data sources. A generalization to more than two data sources with known cross-correlations is given in References [19,20,21,22]. A unified fusion rule for centralized, distributed and hybrid fusion architectures with complete prior information was proposed in References [20,64]. A fusion method for discrete multi-rate independent systems based on multi-scale theory was proposed in Reference [65], where the sampling rate ratio between the local estimates is assumed as a positive integer. Distributed fusion estimation for the case of asynchronous systems with correlated noises was studied in References [66,67,68]. Some authors have also explored learning based approaches for multisensor data fusion [4,6,7,69,70,71]. While Kalman filter and Bayesian formulation rely on known statistics for data fusion, learning based approaches learn the statistical model of the uncertainty from incoming data. In Reference [7], multi-feature fusion method is used for visual recognition in a multimedia application. A fusion framework for multi-rate multisensor linear systems based on a neural network was proposed in Reference [69]. The framework reformulates the multi-rate multiple systems into a single multisensor system with the highest sampling rate and effectively fuse the local estimates using neural network. A neural network based multisensor data fusion is compared with conventional methods in References [72,73] with superior fusion performance. However, learning based approaches are limited with the requirement of a large amount of data for training. Interested readers can refer to References [50,52] for more general perspectives and approaches to multisensor data fusion. 

Given n sensor estimates $\left({\hat{x}}_{1},P_{1}\right),\left({\hat{x}}_{2},P_{2}\right),\ldots ,\left({\hat{x}}_{n},P_{n}\right)$ with exact cross-correlation $P_{ij},i,j=1,\ldots ,n$, the fused mean and covariance can be written as [19,20,21,22],

(9) $${\hat{x}}_{f}=(H^{T}P^{-1}H{)}^{-1}H^{T}P^{-1}\hat{x}$$

(10) $$P_{f}=(H^{T}P^{-1}H{)}^{-1}$$

with

$$\hat{x}=\left[\begin{array}{c} {\hat{x}}_{1}\\ \begin{array}{c} {\hat{x}}_{2}\\ \vdots \\ {\hat{x}}_{n} \end{array} \end{array}\right],\;P=\left[\begin{array}{cc} P_{1} & \begin{array}{ccc} P_{12} & \ldots & P_{1n} \end{array}\\ \begin{array}{c} P_{12}^{T}\\ \vdots \\ P_{1n}^{T} \end{array} & \begin{array}{ccc} \begin{array}{c} P_{2}\\ \vdots \\ \ldots \end{array} & \begin{array}{c} \ldots \\ \ddots \\ \ldots \end{array} & \begin{array}{c} \vdots \\ \vdots \\ P_{nn} \end{array} \end{array} \end{array}\right],\;H=\left[\begin{array}{c} I_{N_{1}}\\ \begin{array}{c} I_{N_{2}}\\ \vdots \\ I_{N_{n}} \end{array} \end{array}\right]$$

where the dimensions of $\hat{x},\;P$ and H are Nn×1, nN×nN and nN×N, respectively. n is the number of sensors and N corresponds to the dimension of the state vector. With full prior information, these fusion rules are proven to be unbiased and optimal in the sense of MMSE. If the estimates are assumed to be independent, that is, $P_{ij}=0,i,j=1,2,\ldots ,n$, then the fused result can be obtained as,

(11) $$P_{f}=\left(\overset{n}{\underset{i=1}{\sum }}P_{i}^{-1}\right){}^{-1}$$

(12) $$x_{f}=P_{f}\left(\overset{n}{\underset{i=1}{\sum }}P_{i}^{-1}{\hat{x}}_{i}\right)$$

In order to employ the fusion rule of (9) and (10), the computation of the cross-covariance $P_{ij}$ is needed. The cross-covariance among local sensor estimates can be calculated as [19,21,22,74],

(13) $$P_{ij}=\left[I-K_{i}H_{i}\right]\left[AP_{ij}^{k-1}A^{T}+BQB^{T}\right]{\left[I-K_{j}H_{j}\right]}^{T}$$

where $K_{i}$ is the Kalman gain of $i_{th}$ local filter and $P_{ij}^{k-1}$ represents the cross covariance of the previous cycle. As seen from (13), the calculation of the cross-covariance needs internal details of the estimator, like the Kalman gain, which may not be available in some cases. An approximation of the cross-covariance in terms of the correlation coefficient can be obtained in such cases [75],

(14) $$P_{ij}=\rho \sqrt{P_{i}P_{j}}$$

**Figure 5:**
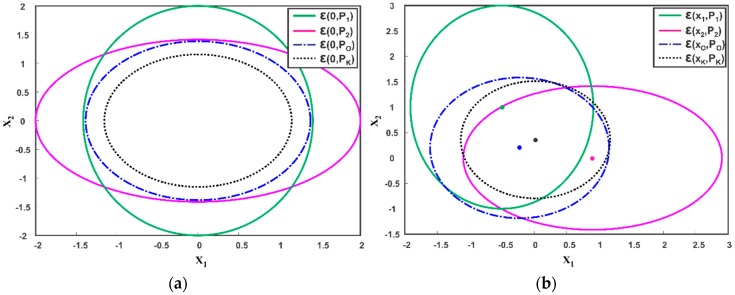
Ellipsoidal Fusion of two Estimates $\varepsilon \left(x_{1},P_{1}\right)$ and $\varepsilon \left(x_{2},P_{2}\right)$ (**a**) Zero Mean (**b**) Non-Zero Mean. Compared to the optimal solution $\varepsilon \left(x_{o},P_{o}\right)$, Kalman filter (KF) yields underestimated results $\varepsilon \left(x_{K},P_{K}\right)$ by ignoring cross-correlation.

An approximation of the cross-covariance in terms of the different correlation components for different components of the state can be computed as,

(15) $$P_{ij}^{nm}=\rho_{nm}\sqrt{P_{i}^{nm}P_{j}^{nm}}$$

where $n,m=1,\ldots ,N_{x}$ with $N_{x}$ as the state dimension. A Monte Carlo simulation can be used to numerically compute the correlation coefficient ρ offline for a specific setup. Figure 5a,b illustrates the effect of the independence assumption on fused covariance and fused mean of two correlated sensor estimates respectively. The optimal fused solution $\varepsilon \left(x_{o},P_{o}\right)$ is obtained using (7) and (8) by incorporating a known cross-correlation. As shown, when KF is employed by assuming zero correlation between the sensor estimates, an underestimated fused covariance and mean is obtained as compared to the optimal fused solution. This severely hampers the accuracy of estimated states and sometimes results in filter divergence.

It is worth noting that the KF/IF provides optimal results in a centralized architecture because the assumption of independence is true. In a distributed fusion architecture, optimality can be achieved by computing and incorporating the exact cross-correlation. Furthermore, addressed fusion algorithms can either be applied independently or jointly to solve complex fusion problems according to fusion architectures and practical demands.

## 4. Fusion under Unknown Correlation

**Figure 6:**
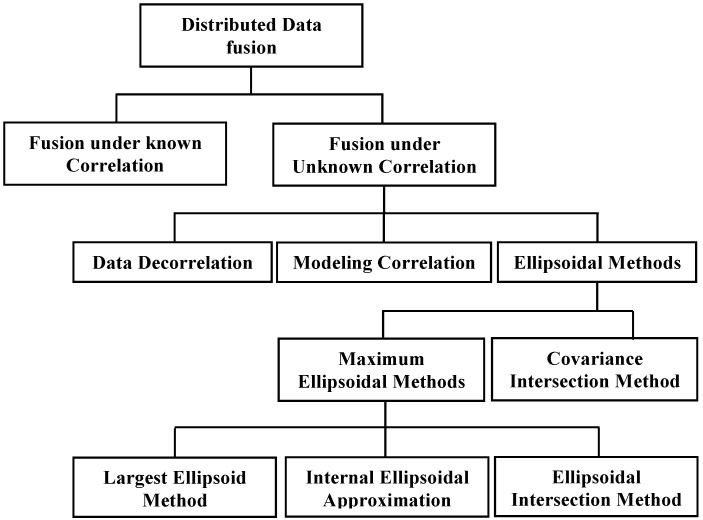
Taxonomy of Fusion under Unknown Correlation.

There are various sources of correlation affecting the state estimation and fusion process in a distributed architecture. Failing to consider the cross-correlation leads to overconfident results and even divergence of the fusion algorithm [9,24]. Nonetheless, due to double counting and the unavailability of internal parameters, it is very difficult to exactly estimate the cross-correlation in a vast distributed sensor network. In some applications, such as in map building, weather forecasting etc., the process model could use hundreds and thousands of states [35]. Maintaining and taking care of cross-correlation is expensive, and it scales quadratically with the number of updates [23]. Therefore, various suboptimal strategies have been devised to provide a fused solution from multiple data sources without the need of an actual cross-correlation. The analysis of fusion under unknown correlation is carried out according to the categorization of Figure 6.

### 4.1. Data Decorrelation

A common cause of cross-correlation in distributed architecture is data incest/rumor propagation/double counting. Double counting happens when the same data follows different or cyclic paths to reach the fusion node [9,13]. An effective way to avoid the data incest issue is to keep the record of estimate updates. References [27,28] propose a method to remove the correlation by explicitly eliminating double counting. The idea is to resolve remote measurements from state estimates of other sensor nodes, store them and use them to update its own state estimate. This way the double counted data is removed before the data is fused. This method assumes a specific network topology to avoid the correlation due to double counting. In References [76,77], a more general solution using graph theoretic algorithms is proposed, which is viable for arbitrary network topologies with variable time delays. However, this is neither scalable nor practical for a large network of sensors [78]. Another approach for decorrelation is measurement reconstruction [25,26,79], where the system noise is artificially adjusted by reconstructing the measurements so that correlation between the sequence of measurements is removed. The remote measurements are reconstructed at the fusion node based on the local sensor estimates. This method is further developed for tracking in clutter [80], Out-of-sequence filtering [81] and non-Gaussian distributions with Gaussian mixture models [82]. However, internal information like Kalman gain, association weights and sensor model information etc. are required to exactly reconstruct the measurements [74,75]. The decorrelation methods result in a compromised fusion performance due to their dependency on empirical knowledge and special analysis for a particular real system. Furthermore, with an increase in the number of sensors, these methods become highly inefficient and impractical.

### 4.2. Modeling Correlation

Although an exact cross-correlation between local estimates in a distributed architecture is difficult to obtain, the properties of the joint covariance matrix put some restriction on the possible cross-correlation. Furthermore, certain applications may provide prior knowledge and constraints on the degree of correlation such that we may infer whether the local estimates are strongly or weakly correlated. In fact, the estimates provided by multiple sensors are neither independent nor exactly dependent, meaning that the cross-correlation is not completely unknown. Thus, the information and knowledge regarding unknown cross-correlations can be exploited to improve the accuracy of the fused solution under unknown correlation. Given two sensor estimates $\left({\hat{x}}_{1},P_{1}\right)$ and $\left({\hat{x}}_{2},P_{2}\right)$, the joint covariance matrix can be written as,

(16) $$P=\left[\begin{array}{cc} P_{1} & P_{12}\\ P_{21} & P_{2} \end{array}\right]$$

where $P_{12}=P_{21}^{T}$ is the cross-correlation between the two estimates. The joint covariance matrix P is positive semidefinite if and only if there is a contraction matrix C such that [83],

$$P_{12}=P_{1}^{1/2}CP_{2}^{1/2}$$

where a contraction matrix C is a matrix with the largest singular value less than or equal to unity. In the case of scalar-valued estimates, the cross-correlation can be computed as,

(17) $$P_{12}=\rho \sqrt{P_{1}P_{2}}$$

where (17) is a function of known individual covariances and a correlation coefficient ρ in the range [−1, 1]. Based on the correlation model (17) an analytic analysis of the BC formula is carried out to give an exact solution for fusion under unknown correlation [29]. A closed-form equation for scalar-valued fusion and an approximate solution for vector valued fusion based on a uniformly distributed correlation coefficient is proposed in Reference [30]. In Reference [84], a tight upper bound for the joint covariance matrix is obtained from individual covariances $P_{1},\;P_{2}$ and the constrained correlation coefficient ρ. Based on bounded correlations, a general method was proposed as the Bounded Covariance Inflation (BCInf) [85] with upper and lower bounds on cross-correlation. The method exploits the available information regarding known independence in the sensor network. The BCInf method was further developed as an Adaptive Bounded Covariance Inflation (ABCInf) by probabilistic and deterministic approaches [86]. An approximate correlation model is adopted for two data sources in high dimensions as [32],

(18) $$P_{12}=\rho C_{1}C_{2}^{T}$$

where ρ is the correlation coefficient and $C_{1}$ is the cholesky decomposition satisfying $P_{1}=C_{1}C_{1}^{T}.$ It is illustrated in Reference [32] that the proposed model ensures the positive semi definiteness of the joint covariance matrix P and agrees with the Canonical Correlation Analysis of multivariate correlation [87]. Based on the correlation model (18), a track association and fusion is carried out in the Maximum Likelihood sense in Reference [31]. In Reference [32], the Cholesky decomposition model of unknown cross-correlation is applied to BC formula, and the fused solution is iteratively approximated based on min-max optimization function for unknown correlation coefficient ρ. Furthermore, a conservative fusion solution is also obtained under the assumption of a uniform distribution of correlation coefficient ρ. In Reference [29], the correlation model (18) was used in BC formula to analytically estimate the maximum bounds of the unknown correlation in track-to-track fusion. The multisensor estimation problem with the assumption of norm-bounded cross-correlation is studied in [88], where the worst-case fused MSE is minimized for all feasible cross-covariances. To utilize some prior information of the cross-covariance, a formulation named allowance of cross-covariance is proposed in Reference [89]. Based on the proposed model an optimal fusion method in the sense of minimizing the worst-case fused MSE by semidefinite programming (SDP) is derived.

For scalar-valued two sensor estimates, the cross-covariance $P_{12}$ is well-defined by the correlation coefficient ρ. Yet, the number of correlation coefficients increases with the number of sensors and the closed-form solution for even scalar-valued estimates becomes difficult. For instance, in the case of three data sources in $R^{1}$ the joint covariance matrix can be written as,

$$P=\left[\begin{array}{ccc} P_{1} & \rho_{12}\sqrt{P_{1}P_{2}} & \rho_{13}\sqrt{P_{1}P_{3}}\\ \rho_{12}\sqrt{P_{1}P_{2}} & P_{2} & \rho_{23}\sqrt{P_{2}P_{3}}\\ \rho_{13}\sqrt{P_{1}P_{3}} & \rho_{23}\sqrt{P_{2}P_{3}} & P_{3} \end{array}\right]$$

Three correlation coefficients can now be noted to represent the dependency among the three data sources and optimizing any function of P in terms of correlation coefficients becomes a daunting task. In general, it is difficult to interpret cross-correlation for more than two data sources in high dimensions. It should also be noted that the general correlation analysis techniques like canonical correlation analysis (CCA) [87] and multivariate linear regression (MLA) [90] have limited use in connection with the cross-correlation among multiple data sources. Since these techniques assess the correlation property when given a vast set of data points. The joint covariance matrix of the multiple data sources, on the other hand, is a block covariance matrix that represents the relationships among the individual states of the sensor and among different sensors.

### 4.3. Ellipsoidal Methods

**Figure 7:**
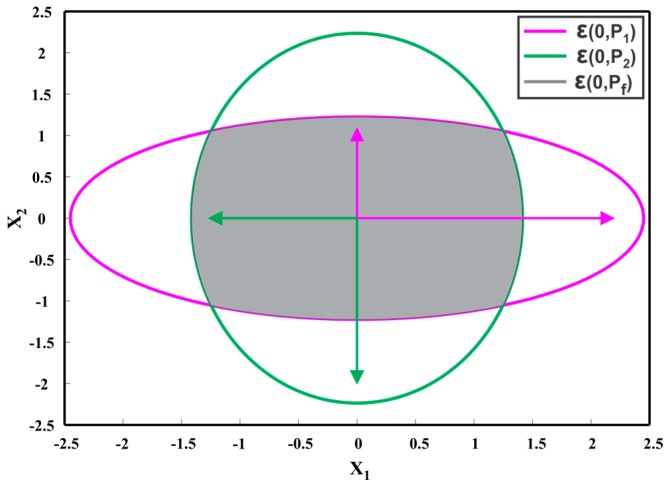
Illustration of fused covariance $P_{f}$ of individual data sources for the correlation coefficient in the range [−1, 1]. The gray area represents all possibilities of a fused covariance.

Suppose that we have two Gaussian sensor estimates $N\left({\hat{x}}_{1},P_{1}\right)$ and $N\left({\hat{x}}_{2},P_{2}\right)$ of the true state x in ${\mathbb{R}}^{2}$. The two data sources are assumed to be correlated with cross covariance matrix $P_{12}$. From (7) and (8), we can observe that the underlying fused covariance and mean of the two data sources is dependent on the unknown cross-covariance $P_{12}$. The given sensor estimates can be represented using an ellipsoid $\mathrm{ellipsoid}\;\varepsilon \left({\hat{x}}_{1},P_{1}\right)\;\mathrm{and}\;\varepsilon \left({\hat{x}}_{2},P_{2}\right)$. Figure 7 depicts the zero mean ellipsoids $\varepsilon \left(0,P_{1}\right)\;\mathrm{and}\;\varepsilon \left(0,P_{2}\right)$, where the length of ellipsoid axes corresponds to the eigenvalues of the respective covariance matrix and the eigenvectors define its orientation. The possible cross covariances between the data sources are bounded [14,33,34,35], which in turn, restricts the possible outcomes of the fused covariance to a bounded set. As shown in Figure 7, for different choices of cross-covariance $P_{12}$, the fused covariance $P_{f}$ will lie inside the intersection of the individual data sources. The goal of the Ellipsoidal Methods (EM) is to find a bounding covariance $P_{EM}$ such that,

(19) $$P_{EM}\geq P_{f}\left(P_{12}\right)$$

for any choice of cross-covariance matrix $P_{12}$. The Ellipsoidal Methods (EM) attempt to provide a fused estimate by approximating the intersection region of the individual ellipsoids. The EM can be further classified into the Covariance Intersection Method (CI), Largest Ellipsoid Method (LE), Internal Ellipsoidal Approximation (IEA) and Ellipsoidal Intersection Method (EI). The three methods, LE, IEA and EI aim for a maximum ellipsoid inside the intersection region of individual ellipsoids, and are termed here as the Maximum Ellipsoidal Methods (ME). The EM are analyzed one by one here.

#### 4.3.1. Covariance Intersection Method

**Figure 8:**
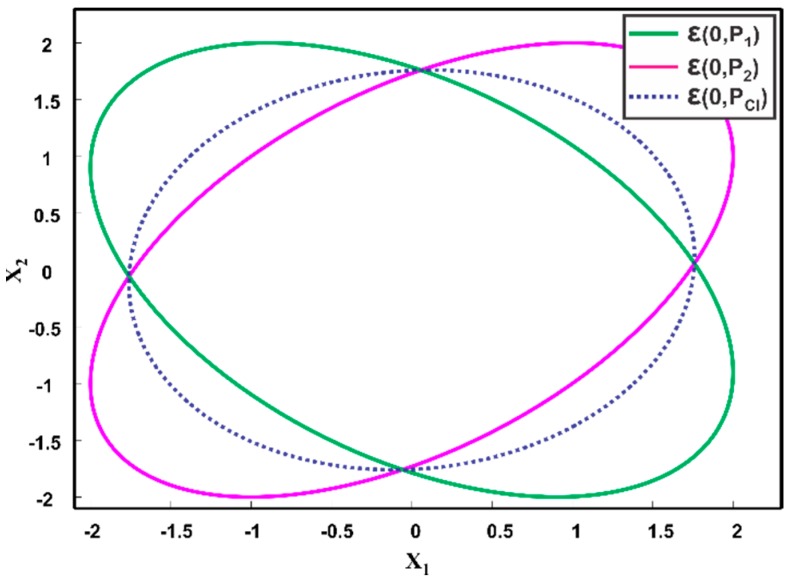
Two estimates at the origin, i.e., $\varepsilon \left(0,P_{1}\right)$, $\varepsilon \left(0,P_{2}\right)$ and their fused result $\varepsilon \left(0,P_{CI}\right)$, provided by Covariance Intersection method.

Covariance Intersection Method (CI) [35] was proposed by Julier and Uhlman for fusion under unknown correlation in a decentralized network. Given two sensor estimates ${\hat{x}}_{1}$ and ${\hat{x}}_{2}$ of the true state x with corresponding covariance matrices $P_{1}$ and $P_{2}$, the CI method can be viewed as a weighted form of the simple convex combination of individual estimates. The algorithm is given by [14,35],

(20) $$x_{CI}=\omega P_{CI}P_{1}{}^{-1}{\hat{x}}_{1}+\left(1-\omega \right)P_{CI}P_{2}{}^{-1}{\hat{x}}_{2}$$

(21) $$P_{CI}{}^{-1}=\omega P_{1}{}^{-1}\;+\;\left(1-\omega \right)P_{2}{}^{-1}$$

where ω∈[0, 1] is a weighting parameter, determined numerically in such a way that the determinant or trace of $P_{CI}$ is minimized. The CI method obtains a consistent fused result without computing the cross-correlation. Figure 8 shows two zero mean estimates as ellipsoids $\varepsilon \left(0,P_{1}\right)$ and $\varepsilon \left(0,P_{2}\right)$. Since, for any possible cross-correlation the fused result lies inside the intersection region of the individual ellipsoids, CI method provides a consistent solution by enclosing the region of the intersection of individual ellipsoids, as depicted in Figure 8.

Since its inception, the CI method has received much attention, and some improvements have been made to enhance the capabilities of the methodology itself while others have focused on its applications in various fields [2,33,34,49,91,92,93,94,95,96,97,98,99,100,101,102,103,104,105,106]. For example, the CI method is generalized as a split CI method [100] to fuse independent as well as dependent information with an unknown degree of correlation. In Reference [97], the CI method is examined with a Chernoff fusion rule, and it is noted that the CI method is suitable for fusing any distributions, and is not limited to Gaussian density function. Meanwhile, CI is used for a non-linear estimation in [107], where the distributions are represented as pseudo-Gaussian densities, while a closed form optimization of CI for low-dimensional matrices was proposed in Reference [103]. In References [108,109], the CI method is studied for track-to-track fusion with memory and without memory. Furthermore, a comparative analysis of CI with different optimal fusion rules is presented in Reference [98]. The CI method is applied in many applications, namely, localization [110,111,112], target tracking [113,114], simultaneous localization and mapping (SLAM) [1,2], image integration [99], NASA MARS rover [101] and spacecraft state estimation [114,115]. 

Although state-of-the-art CI method has its own disadvantages including: (1) requirement of a nonlinear iterative optimization and (2) it overestimates the intersection region of individual covariances, resulting in a degradation of the estimation performance. For the sake of computational efficiency, approaches to directly compute the weights based on the determinants of individual covariances have been proposed [91,92] at the expense of further performance degradation without taking the relative orientation of individual covariances into account. Different optimization criteria for weight computation based on information theory [93,94] as well as set theory [95] have been proposed for computational efficiency. To avoid the computational cost of the CI method for more than two sensors, a sequential covariance intersection (SCI) [96] is presented. The SCI method reduces the multidimensional non-linear optimization problem of CI into many one-dimensional non-linear functions by sequentially applying the CI method of two sensors to n sensors. A proof that CI method results in a minimum consistent covariance bound for two sensors is given in Reference [104]. Recently an Inverse Covariance Intersection (ICI) [105] method based on the common information of two sensors was proposed, which results in a tighter estimate than with the CI method.

#### 4.3.2. Maximum Ellipsoidal Methods

**Figure 9:**
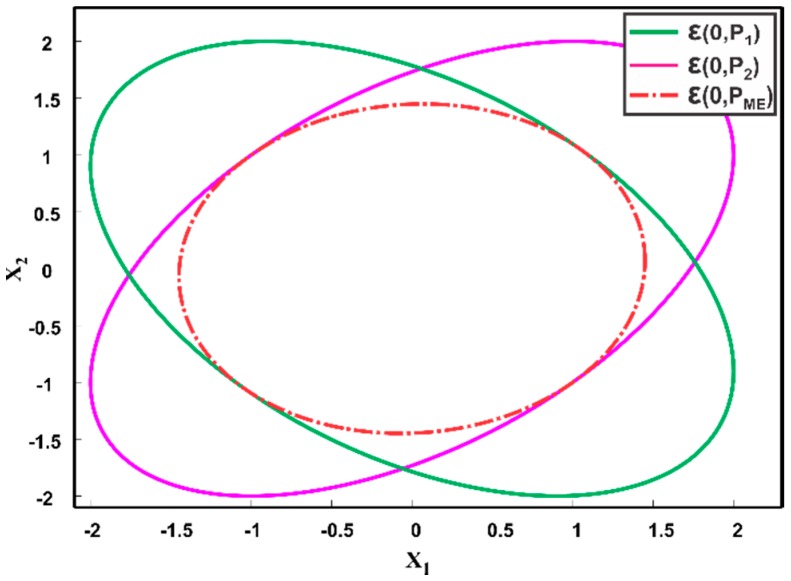
Two estimates at the origin, i.e., $\varepsilon \left(0,P_{1}\right)$, $\varepsilon \left(0,P_{2}\right)$ and the aimed fused result $\varepsilon \left(0,P_{ME}\right)$, of Maximum Ellipsoidal methods.

Contrary to the CI method which yields a minimum overestimation of the intersection region of individual covariances, the Maximum Ellipsoidal Methods (ME), that is, LE [36], IEA [37,38] and EI [39] sought a maximum ellipsoid inside the intersection region of individual covariance ellipsoids as shown in Figure 9. Since the fused covariance for any possible choice of cross-correlation lies inside the intersection of individual ellipsoids, the ME methods attempt to obtain a maximum ellipsoid inside the region of the intersection. Although aiming for a common objective, the ME methods follow different approaches from each other, thus resulting in subtle differences in the computation of the fused mean and covariance. The ME methods are analyzed one by one below.

##### Largest Ellipsoid Method

To avoid an overestimation of the CI, the Largest Ellipsoid Method [36] provides the largest ellipsoid inside the intersection of two individual ellipsoids by manipulating their orientation. Assuming two estimates ${\hat{x}}_{1}$ and ${\hat{x}}_{2}$ with covariances $P_{1}$ and $P_{2}$ respectively. The two covariances are transformed by a transformation matrix $T_{r}$ as,

$$P_{1}^{r}=\;T_{r}\;P_{1}T_{r}^{T},\;P_{2}^{r}=\;T_{r}\;P_{2}T_{r}^{T}$$

where $T_{r}={\left[e_{1}^{T},e_{2}^{T},\ldots ,e_{n}^{T}\right]}^{T}$ is the eigenvector matrix of $P_{1}$. A second scaling transformation is performed by $\;T_{s}$ as,

$$P_{1}^{sr}=\;T_{s}P_{1}^{r}T_{s}^{T}=\;T_{s}\;T_{r}\;P_{1}T_{r}^{T}T_{s}^{T}$$

with

$$T_{s}=diag\left(1,\sqrt{\frac{\lambda_{1_{1}}}{\lambda_{1_{2}}}},\ldots ,\sqrt{\frac{\lambda_{1_{1}}}{\lambda_{1_{n}}}}\right)$$

where $\lambda_{1_{i}}$ is the $i^{th}$ eigenvalue of $P_{1}^{r}$. This scaling operation transform the ellipsoid $P_{1}$ into a sphere with all eigenvalues of $P_{1}^{sr}$ being equal. Similarly, the second ellipsoid is transformed as,

$$P_{2}^{sr}=\;T_{s}\;T_{r}\;P_{2}T_{r}^{T}T_{s}^{T}$$

The intersection of the two ellipsoids $P_{1}^{sr}$ and $P_{2}^{sr}$ in the transformed space is computed as,

$$E^{sr}=EDE^{T}$$

where $E={\left[e_{1}^{T},e_{2}^{T},\ldots ,e_{n}^{T}\right]}^{T}$ is the eigenvector matrix of $P_{2}^{sr}$ and $D=diag\left(k_{1},k_{2},\ldots ,k_{n}\right)$ with $k_{i}=\min\left(\lambda_{1_{i}},\lambda_{2_{i}}\right).$ The corresponding largest ellipsoid is transformed back to original space by an inverse transformation as,

(22) $$P_{LE}=T_{r}^{-1}T_{s}^{-1}E^{sr}T_{s}^{-T}T_{r}^{-T}$$

The fused mean $x_{LE}$ of the two data sources is calculated using the simple convex equation of KF,

(23) $$P_{K}^{-1}x_{LE}=P_{1}^{-1}{\hat{x}}_{1}+P_{2}^{-1}{\hat{x}}_{2}$$

where $P_{K}^{-1}=P_{1}^{-1}+P_{2}^{-1}$.

Although, the LE method for fused covariance results in the largest ellipsoid inside the intersection of the individual ellipsoids, the computation of the fused mean is incorrect. Because calculation of the fused mean is based on the independence assumption of KF and does not consider the cross-correlation, which may lead to inconsistent results. To ensure the consistency and optimality in multisensor data fusion, the fused covariance, as well as the correct calculation of the fused mean, is important. 

##### Internal Ellipsoidal Approximation

To fill the gap in the LE Method, an Internal Ellipsoidal Approximation Method (IEA) [37,38, 116] was proposed which provides an internal approximation of the region of intersection of the individual ellipsoids. The fused mean and covariance of the algorithm are written as,

(24) $$x_{IEA}=(\omega_{1}P_{1}{}^{-1}+\omega_{2}P_{2}{}^{-1}{)}^{-1}\left(\omega_{1}P_{1}{}^{-1}{\hat{x}}_{1}+\omega_{2}P_{2}{}^{-1}{\hat{x}}_{2}\right)$$

(25) $$P_{IEA}=\left(1-\hat{x}{}_{1}^{T}P_{1}{}^{-1}{\hat{x}}_{1}-\hat{x}{}_{2}^{T}P_{2}{}^{-1}{\hat{x}}_{2}+\hat{x}{}_{IEA}^{T}P_{IEA}{}^{-1}{\hat{x}}_{IEA}\right)\left(\omega_{1}P_{1}{}^{-1}+\omega_{2}P_{2}{}^{-1}\right){}^{-1}$$

where

(26) $$\omega_{1}=\frac{1-\min\left(1,\beta_{2}\right)}{1-\min\left(1,\beta_{1}\right)\min\left(1,\beta_{2}\right)}$$

(27) $$\omega_{2}=\frac{1-\min\left(1,\beta_{1}\right)}{1-\min\left(1,\beta_{1}\right)\min\left(1,\beta_{2}\right)}\;$$

where $0\leq \omega_{1},\omega_{2}\leq 1$ and $\beta_{1}$ and $\beta_{2}$ are computed based on the optimization of the Quadratic programming problem as follows,

(28) $$\beta_{1}=\underset{x^{T}P_{2}{}^{-1}x=1}{\min}x^{T}P_{1}{}^{-1}x$$

(29) $$\beta_{2}=\underset{x^{T}P_{1}{}^{-1}x=1}{\min}x^{T}P_{2}{}^{-1}x$$

Nonlinear optimization methods like Newton or Lagrange multipliers can be used to compute the values of $\beta_{1}$ and $\beta_{2}$. By additional manipulation, the Quadratic Constrained Quadratic Problem (QCQP) of (28) and (29) can be transformed to a much simpler form, resulting in a direct computation of unknown variable x. Based on the definition of $P_{1}$ and $P_{2}$ as positive semidefinite matrices we can write,

$$P_{2}=ED^{1/2}D^{1/2}E^{T}$$

where D is the eigenvalue matrix and E is the respective eigenvector matrix. Using $y=D^{-1/2}E^{T}x$, we can rewrite (28) in terms of y as,

(30) $$\beta_{1}=\underset{{\left\|y\right\|}_{2}^{2}=1}{\min}y^{T}\left(D^{\frac{1}{2}}E^{T}P_{1}{}^{-1}ED^{\frac{1}{2}}\right)y$$

Hence,

$$y^{T}\left(D^{\frac{1}{2}}E^{T}P_{1}{}^{-1}ED^{\frac{1}{2}}\right)y\geq \lambda_{min}\left(D^{\frac{1}{2}}E^{T}P_{1}{}^{-1}ED^{\frac{1}{2}}\right){\left\|y\right\|}_{2}^{2}$$

Then $y_{min}$, the normalized eigenvector corresponding to the minimum eigenvalue of $\left(D^{\frac{1}{2}}E^{T}P_{1}{}^{-1}ED^{\frac{1}{2}}\right)$ is a solution to (30). Subsequently, x can be obtained as,

$$x=ED^{1/2}y_{min}$$

The value of x can be used in (28) to obtain $\beta_{1}$. A similar approach can be followed to calculate $\beta_{2}$. The computed values of $\beta_{1}$ and $\beta_{2}$ can then be used in (26) and (27) to compute the weights $\omega_{1}$ and $\omega_{2}$. Based on the values of $\beta_{1}$ and $\beta_{2}$, the IEA method provides a relationship between two ellipsoids as [37,116],

1. If $\beta_{1}\geq 1,\;\beta_{2}\leq 1,$ then $\omega_{1}=1,\;\omega_{2}=0$, $\varepsilon \left(0,P_{1}\right)\subseteq \varepsilon \left(0,P_{2}\right)$ and $\varepsilon \left(x_{IEA},P_{IEA}\right)=\varepsilon \left(x_{1},P_{1}\right)$
2. If $\beta_{1}\leq 1,\;\beta_{2}\geq 1,$ then $\omega_{1}=0,\;\omega_{2}=1,$ $\varepsilon \left(0,P_{1}\right)⊇\varepsilon \left(0,P_{2}\right)$ and $\varepsilon \left(x_{IEA},P_{IEA}\right)=\varepsilon \left(x_{2},P_{2}\right)$
3. If $\beta_{1}\leq 1,\;\beta_{2}\leq 1,$ then $0<\omega_{1},\;\omega_{2}<1,$ $\varepsilon \left(0,P_{1}\right)\cap \varepsilon \left(0,P_{2}\right)\neq \phi$

Although the IEA method aims for an approximation of the intersection region of individual ellipsoids, the method lacks a strong mathematical foundation and is based on heuristics.

##### Ellipsoidal Intersection Method

Ellipsoidal Intersection (EI) Method [39] solves the problem of fusion under unknown correlation by computing the fused mean and covariance based on the mutual and exclusive information of two data sources. Given two sensor estimates $\left({\hat{x}}_{1},P_{1}\right)$ and $\left({\hat{x}}_{2},P_{2}\right)$, it is assumed that they can be represented by three mutually uncorrelated estimates $\left(\hat{a},A\right),\;\left(\hat{b},B\right)$ and (Υ,Γ) as [117],

(31) $$P_{1}=\left(A^{-1}+\Gamma^{-1}\right){}^{-1},{\hat{x}}_{1}=P_{1}\left(A^{-1}\hat{a}+\Gamma^{-1}\Upsilon \right)$$

(32) $$P_{2}=\left(B^{-1}+\Gamma^{-1}\right){}^{-1},{\hat{x}}_{2}=P_{2}\left(B^{-1}\hat{b}+\Gamma^{-1}\Upsilon \right)$$

Hence, both sensor estimates share the common estimate (Υ,Γ) . By using mutual and exclusive information, the fused mean and covariance of the algorithm is written as,

(33) $$P_{EI}={\left(P_{1}{}^{-1}\;+\;B^{-1}\right)}^{-1},\;x_{EI}=P_{EI}\left(P_{1}{}^{-1}{\hat{x}}_{1}+B^{-1}\hat{b}\right)$$

Substituting the results of (32) in (33) gives the fused covariance $P_{EI}$ and fused mean $x_{EI}$ as,

(34) $$P_{EI}=\left(P_{1}{}^{-1}\;+\;P_{2}{}^{-1}-\Gamma^{-1}\right){}^{-1}$$

(35) $$x_{EI}=P_{EI}\left(P_{1}{}^{-1}{\hat{x}}_{1}+P_{2}{}^{-1}{\hat{x}}_{2}-\Gamma^{-1}\Upsilon \right)$$

The formulation of (34) and (35) implies that first the estimates $\left({\hat{x}}_{1},P_{1}\right)$ and $\left({\hat{x}}_{2},P_{2}\right)$ are fused, followed by subtraction of the common estimate (Υ,Γ). The mutual covariance Γ is chosen such that the mutual information between the two data sources is maximized. Using eigenvalue decomposition, we can write,

$$P_{1}=E_{1}D_{1}E_{1}{}^{T},\;\mathrm{and}\;Q_{2}D_{2}Q_{2}{}^{T}=D_{1}^{-0.5}E_{1}^{T}P_{2}E_{1}D_{1}^{-0.5}$$

Then, the maximum mutual information can be calculated as,

(36) $$\Gamma =E_{1}D_{1}^{0.5}Q_{2}D_{\Gamma }Q_{2}{}^{T}D_{1}^{0.5}E_{1}{}^{-1}$$

where

$${\left(D_{\Gamma }\right)}_{ij}=\{\begin{array}{c} \max\left(D_{2},1\right)\;\;if\;i=j\\ 0\;\;\;\;\;\;\;\;\;\;\;if\;i\neq j\; \end{array}$$

Similarly, the mean value of the mutual information can be computed as,

(37) $$\Upsilon =\left(P_{1}{}^{-1}+P_{2}{}^{-1}-2\Gamma^{-1}+2\eta I\right){}^{-1}\left(\left(P_{2}{}^{-1}-\Gamma^{-1}+\;\eta I\right){\hat{x}}_{1}+\left(P_{1}{}^{-1}-\Gamma^{-1}+\eta I\right){\hat{x}}_{2}\right)$$

where the term η is added such that ($P_{i}{}^{-1}-\Gamma^{-1}$) should be positive definite rather than positive semi-definite. The value of η is selected as follow,

$$\eta =\{\begin{array}{c} 0\;\;\;\;\;\;\;\;\;\;\;\;\;\;\;\;if\;\left|H\right|\neq 0\\ s\ll \lambda_{+}\left(H\right)\;\;\;\;\;\;\;if\;\left|H\right|=0\; \end{array}$$

where H is defined as $H=P_{1}{}^{-1}+P_{2}{}^{-1}-2\Gamma^{-1}$ and $\lambda_{+}\left(H\right)\in R_{+}$ is defined as the smallest non-zero eigenvalue of H.

A relation between the cross-covariance $P_{12}$ and mutual information Γ of $P_{1}$ and $P_{2}$ is given as [105],

(38) $$P_{12}=P_{1}\Gamma^{-1}P_{2}$$

Based on (38), a decentralized fused solution for two sensor estimates known as inverse covariance intersection (ICI) is proposed in Reference [105]. This method provides a tighter solution than CI for all admissible common information Γ. The concept of common information is also used in the channel filter [12] and its nonlinear counterpart [118]. In Reference [119], the performance of the EI method is assessed for various real-life scenarios like the absence of observability, non-linearity of the process model and situations where the computational requirement is different for different nodes. For fusion of scalar-valued estimates, the fused solution provided by EI is equal to that of CI method.

**Example.**  *Consider an illustrative example for comparative analysis of EM with the following two sensor estimates,*

$${\hat{x}}_{1}=\left[\begin{array}{c} 1\\ 2 \end{array}\right],\;P_{1}=\left[\begin{array}{cc} 4 & 1.8\\ 1.8 & 3.5 \end{array}\right],\;{\hat{x}}_{2}=\left[\begin{array}{c} 0.8\\ 1.3 \end{array}\right],\;P_{2}=\left[\begin{array}{cc} 4.5 & 0.5\\ 0.5 & 2.7 \end{array}\right]$$

**Figure 10:**
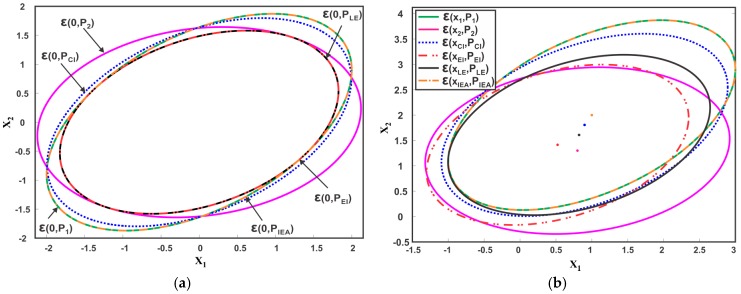
Two estimates $\varepsilon \left(x_{1},P_{1}\right)$ and $\varepsilon \left(x_{2},P_{2}\right)$ and their fused result provided by CI and ME methods, where three instances of ME are considered (**a**) Zero Mean (**b**) Non-Zero Mean.

The weights of the CI method are determined by minimizing the determinant of the fused covariance, that as, $\underset{\omega +\left(1-\omega \right)=1\;}{\min}\det\left(P_{CI}\right)$. The Matlab function ‘fminbnd’ is used to compute the weights and are then used in (20) and (21) to compute the fused mean and fused covariance of the CI method. For IEA, the parameters $\beta_{1}$ and $\beta_{2}$ are computed using (30) and subsequently, the weights $\omega_{1}$ and $\omega_{2}$ are computed from (26) and (27) respectively. The weights are then used to compute the fused result. The fused covariance and mean of the LE and EI method are calculated using (22), (23) and (34), (35) respectively. The eigenvalue decomposition of the ME methods is done using the standard ‘eig’ function of Matlab. Table 1 summarizes the computed fused mean and covariance of different EM. The average computation time of each method for 10,000 runs is also given in Table 1. Figure 10a,b depicts the fused covariance ellipsoids of the different EM. The CI method can be noted to provide a minimum overestimate of the intersection region of the individual data sources. The IEA method chooses the first sensor estimate as the fused result despite the fact that $\varepsilon \left(0,P_{1}\right)⊈\varepsilon \left(0,P_{2}\right)$. The LE and EI result in a maximum covariance ellipsoid inside the intersection region. Although aiming for the same goal, the three ME methods differ from each other. For instance, the fused covariance provided by EI and LE is exactly the same while the fused covariance provided by IEA differ from LE and EI methods in this case. On the other hand, the fused mean provided by all three ME methods are different as noted from Figure 10b and Table 1. 

The CI method provides a consistent fused solution for two estimates based on (19), that is, $P_{CI}-P_{f}$ is always positive semi-definite. This can also be observed from Figure 10a, where CI method generate a tight bound on the intersection region, thus ensuring consistency for any choice of cross-correlation. Although consistent, the CI results are conservative with the possibility of much less informative fused estimates. On the other hand, the LE and EI methods result in a largest ellipsoid inside the region of intersection. However, the methods may become inconsistent with $P_{LE},P_{EI}≱P_{f}$, for some choices of known cross-covariance $P_{12}$. The EI method yields less conservative results than CI and may perform better when the local sensor estimates are weakly correlated.

**Figure 11:**
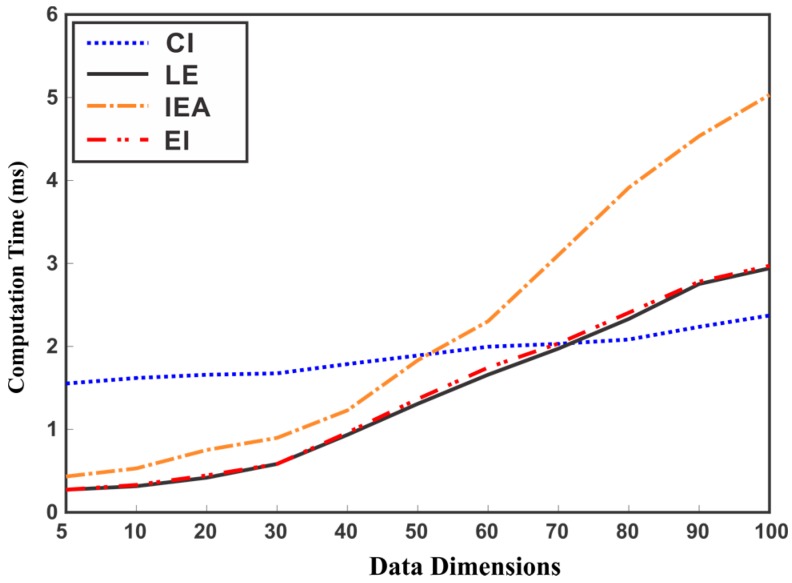
Comparison of CI and ME methods in terms of computation time for different dimensions of data.

It can be observed from Table 1 that the CI method incurs high computational cost as compared to the other methods. To observe the effect of data dimension on the computation time of EM methods, we randomly generated data with different dimensions for evaluation. Figure 11 depicts the average computation time for 10,000 runs of each method for fusing two data sources of increasing dimension. Although, the ME methods perform efficiently for low dimensions of data, these methods may become inefficient with the increase in the dimensions of data sources as seen from Figure 11. 

#### 4.3.3. Analysis of Ellipsoidal Methods for Three Sensors

In some situations, more than two sensors may provide an estimate of a particular state in a distributed sensors system. The role of the data fusion framework is to provide a consistent and minimum variance fused solution when more than two sensors are involved. The framework of all the three ME methods are devised for fusing two sensors only. Conservative solutions can be achieved for fusion of more than two sensors by sequentially applying the ME methods in a decentralized fashion similar to SCI [96]. The CI method, on the other hand, provides a generalization to n sensors [49]. The CI method computes an estimate $P_{CI}$ for n sensors by combining the individual covariances $P_{i},i=1,\ldots ,n$ with scalars $\omega_{i},$ such that, $\sum_{i=1}^{n}\omega_{i}=1$ is retained. The fused mean and covariance estimate for n sensor estimates are then obtained as,

(39) $$x_{CI}=P_{CI}\left(\omega_{1}P_{1}^{-1}{\hat{x}}_{1}+\omega_{2}P_{2}^{-1}{\hat{x}}_{2}+\ldots +\omega_{n}P_{n}^{-1}{\hat{x}}_{n}\right)$$

(40) $$P_{CI}=\left(\omega_{1}P_{1}^{-1}+\omega_{2}P_{2}^{-1}+\ldots +\omega_{n}P_{n}^{-1}\right){}^{-1}$$

However, a simple example reveals that the minimum overestimate of CI for more than two sensors does not hold.

**Example.**  *Consider an illustrative example with the following three sensor estimates,*

$${\hat{x}}_{1}=\left[\begin{array}{c} 0\\ 0 \end{array}\right],\;P_{1}=\left[\begin{array}{cc} 0.5 & 0\\ 0 & 8 \end{array}\right],\;{\hat{x}}_{2}=\left[\begin{array}{c} 0\\ 0 \end{array}\right],\;P_{2}=\left[\begin{array}{cc} 6.1250 & 3.2476\\ 3.2476 & 2.3750 \end{array}\right],\;\;{\hat{x}}_{3}=\left[\begin{array}{c} 0\\ 0 \end{array}\right],P_{3}=\left[\begin{array}{cc} 6.1250 & -3.2476\\ -3.2476 & 2.3750 \end{array}\right]$$

**Figure 12:**
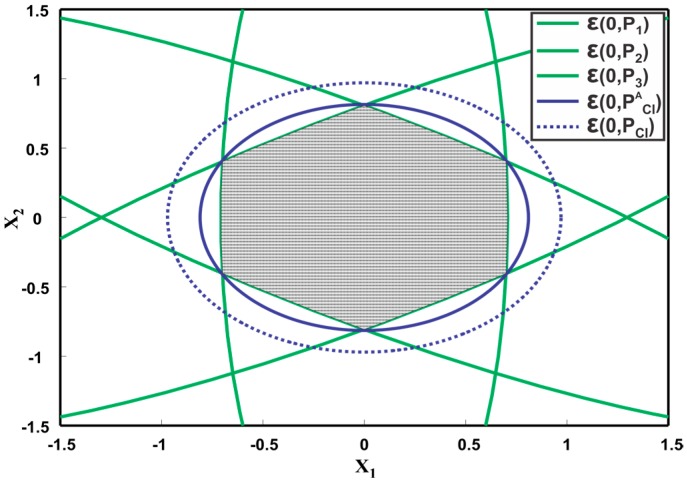
Illustration of three ellipsoids $\varepsilon \left(0,P_{1}\right),\varepsilon \left(0,P_{2}\right),\;\varepsilon \left(0,P_{3}\right)$ and their fusion result $\varepsilon \left(0,P_{\mathrm{CI}}\right),$ provided by CI method. The figure also shows the actual fused result $\varepsilon \left(0,P_{CI}^{A}\right)$ for CI.

**Figure 13:**
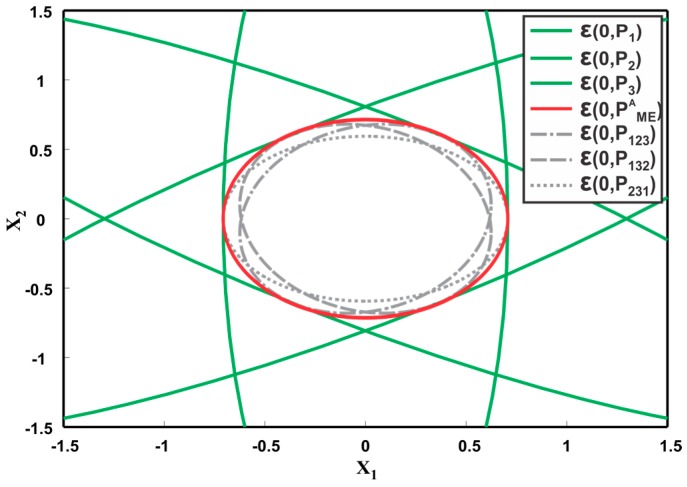
Illustration of three ellipsoids $\varepsilon \left(0,P_{1}\right),\varepsilon \left(0,P_{2}\right),\;\varepsilon \left(0,P_{3}\right)$ and their fusion result provided by ME methods. The figure also shows the actual fused result $\varepsilon \left(0,P_{ME}^{A}\right)$ for ME methods.

Figure 12 depicts the corresponding covariance ellipsoids of the three sensors. The fused covariance of the three sensors for different values of correlation lies inside the hexagonal intersection area of the three ellipsoids. By definition, the CI method should provide a tight overestimation of the hexagonal intersection region as shown in Figure 12 as $\varepsilon \left(0,P_{CI}^{A}\right).$ However, trace minimization of $P_{CI}=\left(\sum_{i=1}^{n}\omega_{i}P_{i}^{-1}\right){}^{-1}$ leads to a larger overestimate than the actual one. This means that the generalization of CI as a minimum tight overestimate for more than two sensors must be different than as proposed in [49]. Figure 13 shows the fused results provided by sequentially applying the ME methods to three sensors. First, the two sensor estimates are fused together, followed by fusion of the third estimate. The fused covariance ellipsoid for three sequences, that is, $P_{123},P_{132}$ and $P_{231}$ are depicted. Consequent of ME methods definition, the fused result for three sensors must be a maximum ellipsoid inside the intersection region $\varepsilon \left(0,P_{EM}^{A}\right)$ as shown in Figure 13. However, the ME methods provide underestimated fused solutions as depicted in Figure 13. It can also be noted that different sequence of fusion result in different fused ellipsoid.

**Remarks.**  *The choice of a fusion method under the assumption of unknown cross-correlation depend on the underlying fusion problem. The data decorrelation methods remove the correlation before fusing the estimates but are limited to small network topologies. It is always preferable to use exact cross-correlation in a distributed fusion architecture to achieve optimality. As such, if there is some prior knowledge of the extent of the correlation, then using that information can improve the estimation accuracy. The CI method can be used to consistently fuse data with unknown correlation. However, the CI results are conservative with the possibility of a much lower accuracy. The EI method can be used to obtain a less conservative solution. Table 2 summarizes the characteristics of various methods for fusion under unknown correlation*.

## 5. Fusion of Inconsistent and Spurious Data

The distributed fusion methodologies discussed above assume that input sensor mean and covariance estimates are consistent. In other words, the covariance provides a good approximation of all disturbances affecting the sensor measurements. However, in reality, uncertainties in sensor measurements may not only come from noise but also from unexpected situations, such as short duration spike faults, sensor glitches, permanent failure or slowly developing failure due to sensor elements [40,41,42]. Since these types of uncertainties are not attributable to the inherent noise, they are difficult to model. Subsequently, the estimates provided by a sensor node in a distributed sensor network may be spurious and inconsistent. Fusing such inconsistent estimates with correct estimates can lead to severely inaccurate results [43]. Hence, a data validation scheme is required to identify and eliminate the sensor inconsistencies before fusion in a distributed architecture. Various methods exist in the literature to tackle the issue of data inconsistency and can be broadly categorized into three groups based on their approach to the problem. These groups of methods are overviewed one by one here.

### 5.1. Model Based Approaches

The model-based approaches, also known as analytical redundancy approaches [45,46] identify functional relationships among the measured states through a mathematical model that can either be developed from the underlying physics or derived directly from the measurements. A residual $r_{k}$ is then generated between the actual sensor output $y_{k}$ and estimated modeled output ${\hat{y}}_{k}$, i.e.,

$$r_{k}=y_{k}-{\hat{y}}_{k}$$

A zero-mean residual, that is, $E\left[r_{k}\right]=0$ mean no fault and deviation of the mean from zero signify presence of fault. In Reference [120], a Nadaraya-Watson statistical estimator and a priori observations are used to validate the sensor measurements. In References [121,122,123], residuals or innovations generated by Kalman filter (KF) were used for faults detection. The faults are identified by statistical tests on the whiteness, mean and covariance of the residuals. A failure detection approach for GPS integrity monitoring system based on KF was proposed in Reference [123]. The idea is to process subsets of the measurements by a bank of auxiliary KFs and use the generated estimate as a reference for failure detection. In Reference [124], the KF prediction was used as a reference to detect inconsistencies in sensor measurements. An adaptive sensor/actuator fault detection and isolation scheme based on KF for an Unmanned Aerial Vehicle (UAV) was proposed in Reference [125]. The method detects faults in the system by applying statistical test on the innovation covariance of KF. The method then adapt the process and measurement noise accordingly to avoid the deterioration of state estimation due to inconsistencies. This method is used in Reference [126] for improving the accuracy of personal positioning systems for outdoor environment. Common tools for evaluating the statistical characteristics of the residuals are generalized likelihood ratio test [127], chi-square test [128] and multiple hypothesis test [46]. Some authors have also proposed Extended KF (EKF) [129,130] and Unscented KF (UKF) [131] based approaches with the advantage of inconsistencies detection in non-linear systems. Multisensor data fusion with fault detection and removal based on Kullback-Leibler Divergence (KLD) for multi-robot system was proposed in Reference [132]. The method computes the KLD between the a priori and posteriori distributions of the Information Filter (IF) and uses Kullback-Leibler Criterion (KLC) thresholding to detect and remove the spurious sensor data. 

Some researchers have also used fuzzy logic [133,134], knowledge-based [135] and neural network (NN) [136,137,138,139] based approaches to identify sensor inconsistencies. In Reference [135] a knowledge-based machine learning approach is used to solve the interference and drift problem caused by sensor aging in E-nose. A probabilistic NN for sensor validation of jet engines was presented in Reference [136]. The network was trained on comprehensive data of faulty and healthy situations generated from an engine performance model. A turbo fan engine was used to evaluate the performance of the network with high success rate of faults identification. As compared to the conventional model based approaches which require bank of estimators for sensor validation, an efficient AI based method was proposed in Reference [137] for fault detection. The method employed a single NN estimator and achieved the same performance as the group of parallel estimators but with much lower computational cost. In Reference [140], the residual of a recurrent neural network (RNN) was used to identify faults in sensor and actuator of non-linear systems. A NN for fault detection in aircraft sensors and actuators was proposed in Reference [139], where EKF was used to update the weights of the neural network. The use of EKF for tuning the weights of neural network result in a fast convergence rate of learning. The method was found to be more accurate and efficient than conventional NN based approach in faults detection. 

The model based approaches can be used by individual sensor nodes in a distributed architecture to validate their own estimates before transmitting it to the fusion center. In addition, it can be also employed at the central node for validating the incoming multisensory data. The disadvantage of the model based approaches is the requirement of explicit mathematical model and prior information for sensor validation which may not be available in some cases. The learning based approaches ease this requirement by learning the statistical characteristics of the system from training data. However, learning based approaches need a large amount of data for training and depend on the accumulated experience and data history of the target system.

### 5.2. Redundancy Based Approaches

In data/hardware/sensor redundancy based approaches, two or more sensors measure the same critical state and then detect as well as isolate the faulty sensors by consistency checking and majority voting [45]. For instance, voter-based fault detection system for multiple sensors subsystems of GPS, inertial navigation system (INS) and Doppler attitude and heading reference system (DAHRS) was presented in Reference [47]. The method is based on the overlap of Gaussian confidence regions of two local sensor estimates in a decentralized system. A sensor voter algorithm to manage three redundant sensors was presented in Reference [141]. Inconsistency detection for hypersonic cruise vehicles (HCVs) based on redundant multisensor navigation systems was proposed in Reference [142]. The system consists of two blocks, where the first block consists of complementary sensors of inertial navigation system (INS) and GPS, and the second block comprises of INS and celestial navigation system (CNS). The method uses chi-square test and sequential probability ratio test (SPRT) to detect inconsistencies in the local sensor estimates of each block before their data is sent to the central node for obtaining a global estimate. Fault detection and isolation application on redundant aircraft sensors based on fuzzy logic and majority voting were proposed in References [143, 144], respectively. Without any prior information, a method to detect spurious sensor data based on Bayesian framework was proposed in Reference [40,41]. The method adds a term to the Bayesian formulation which has the effect of increasing the posterior distribution when measurement from one of the sensors is inconsistent with respect to the other. Gaussian likelihood function of a state X in the presence of measurements $z_{1}$ and $z_{2}$ from a pair of sensors can be written as,

(41) $$p\left(Z=z_{n}|X=x\right)=\frac{1}{\sigma_{n}\sqrt{2\pi }}e^{\left\{\frac{-{\left(x-z_{n}\right)}^{2}}{2\sigma_{n}^{2}}\right\}}\;n=1,2$$

The posterior fused mean and covariance can be computed as,

$$x_{f}=\frac{\sigma_{2}^{2}}{\sigma_{1}^{2}+\sigma_{2}^{2}}z_{1}+\frac{\sigma_{1}^{2}}{\sigma_{1}^{2}+\sigma_{2}^{2}}z_{2},\;\sigma_{f}^{2}=\left[\sigma_{1}^{-2}+\sigma_{2}^{-2}\right]{}^{-1}$$

The method developed a modified Bayesian (MB) formulation as,

(42) $$p\left(X=x|Z=z_{n}\right)\propto \frac{1}{\sigma_{1}\sqrt{2\pi }}e^{-\frac{{\left(x-z_{1}\right)}^{2}}{2\sigma_{1}^{2}f}\;}\;\times \frac{1}{\sigma_{2}\sqrt{2\pi }}e^{-\frac{{\left(x-z_{2}\right)}^{2}}{2\sigma_{2}^{2}f}\;}$$

where $f=\left\{\frac{m^{2}}{m^{2}-{\left(z_{1}-z_{2}\right)}^{2}}\right\}$ and m represent the maximum expected difference between the sensor readings. The factor f depends on the squared difference between the measurements and has the effect of increasing or decreasing the variance of the posterior fused distribution as compared to individual sensor variances. Thus, the MB framework is capable of determining if fusing two measurements would lead to an increase or decrease in posterior distribution variance. Subsequently, a decision to fuse or not can be made based on an increase or a decrease in the posterior variance. In References [43,145], the MB framework along with Kalman filtering is applied to improve the accuracy of robotic position estimation in the presence of inconsistencies. In Reference [8], a fault-tolerant multisensor perception system was presented for mobile robot localization with redundant parallel blocks. Where each block consists of duplicate sensors and fusion block. The idea is to compare sensors measurements of the redundant sensors from each block as well as the KF fused result of individual block to detect inconsistencies. 

Redundancy based approaches may fail if multiple sensors could fail simultaneously. This is possible due to the fact that redundant sensors operate in the same working environment and thus tend to have similar usage life expectations. In Reference [146], a combination of model based approach and majority voting is used to remove modeled and unmodeled faults in a target tracking scenario. Similarly, a hybrid of data redundancy and analytic redundancy based on unscented and extended Kalman filter is proposed in References [147,148] respectively.

### 5.3. Fusion Based Approaches

Some authors also explored fusion of inconsistent sensor estimates within the Bayesian probabilistic framework. For instance, Uhlman proposed a Covariance Union (CU) [49] to consistently fuse spurious data coming from multiple sources. The CU method unifies two or more sensor estimates that are inconsistent. Given n local estimates $\left({\hat{x}}_{1},P_{1}\right)$, $\left({\hat{x}}_{2},P_{2}\right)\ldots \left({\hat{x}}_{n},P_{n}\right)$, the CU method provides a unioned estimate $\left({\hat{x}}_{u},P_{u}\right)$, which is consistent with all of the estimates as long as one of the estimate $\left({\hat{x}}_{i},P_{i}\right)$ is consistent. The CU constraint is,

(43) $$P_{u}\geq P_{1}+\left(u-{\hat{x}}_{1}\right)\left(u-{\hat{x}}_{1}\right){}^{T}\;P_{u}\geq P_{2}+\left(u-{\hat{x}}_{2}\right)\left(u-{\hat{x}}_{2}\right){}^{T}\;\vdots \;P_{u}\geq P_{n}+\left(u-{\hat{x}}_{n}\right)\left(u-{\hat{x}}_{n}\right){}^{T}$$

For a pair of estimates, a close form representation of CU fused covariance can be obtained. Define:

$$P_{1}=E_{1}D_{1}E_{1}^{T}$$

**Figure 14:**
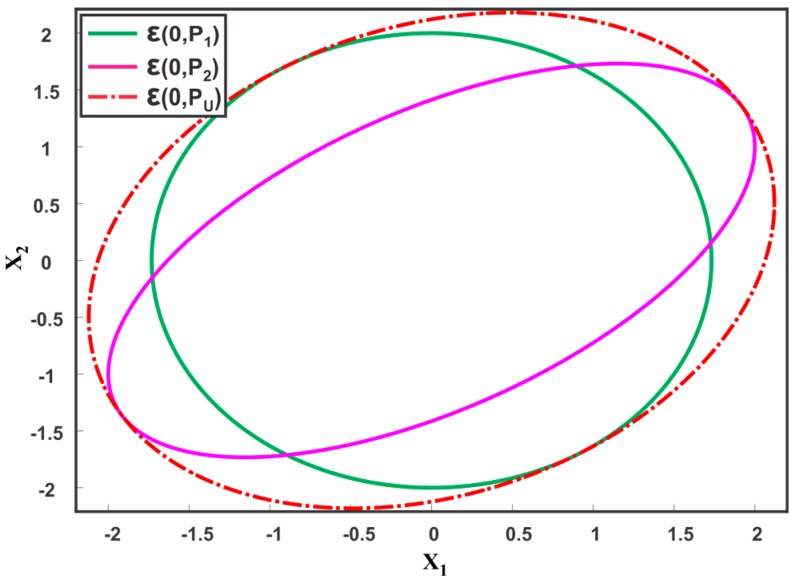
Illustration of two ellipsoids $\varepsilon \left(0,P_{1}\right),\varepsilon \left(0,P_{2}\right)$ and their consistent fused result $\varepsilon \left(0,P_{U}\right)$, provided by the CU method.

Then

$$I=TP_{1}T^{T}\;and\;P_{2}^{\prime }=TP_{2}T^{T}=E_{2}D_{2}E_{2}^{T}$$

where $T=D_{1}^{-1/2}E_{1}^{T}$ and I is the identity matrix. Then, we can write

$$P_{u}=E_{1}D_{1}^{1/2}E_{2}max\left(D_{2},I\right)E_{2}^{T}D_{1}^{1/2}E_{1}^{T}$$

where max is the element wise maximum value of $D_{2}$ and I matrices. Figure 14 shows the merging of two coincident estimates by CU. The union fused result for multiple sensor estimates can be obtain by solving the CU constraints of (43) by numerical optimization [149]. In References [51,150], the CU method is explored to consistently fuse more than two sensor estimates. To ensure consistency for more than two estimates, the CU method should be collectively applied rather than pairwise recursively [150]. Furthermore, an implementation of the CU algorithm in MATLAB and C is developed in Reference [150]. However, the implementation incurs a high computational cost and is not practical for real-time applications. Proof that the CU method provides a minimum enclosing ellipsoid for fusion of local estimates is given in Reference [151]. A Generalized Covariance Union (GCU) to merge multiple hypotheses in tracking applications is presented in Reference [48]. The GCU method provides tighter estimates than CU by exploiting the hypothesis probability bounds. The method reduces to CU when hypothesis probability is absent and to standard mixture reduction (SMR) methods when the hypothesis probability is exactly known. The CU method is studied for navigation [152] and in comparison with other track-to-track fusion algorithms [129], and is shown to perform well in the presence of inconsistencies. A hybrid of the CI and CU method for network-centric data fusion is shown to be highly flexible and resilient against corrupted sensor data [153]. However, the CU method incurs a high computational cost and results in an inappropriately large conservative fused solution.

**Remarks.**  *It should be noted that, to ensure consistency in distributed data fusion, the effect of spurious data needs to be taken into consideration in addition. To this end, methods for identifying spurious data and managing consistency under spurious data, either by removing spurious data or enlarging fused covariance are introduced. The choice of fault-tolerant methods for distributed data fusion depends upon the underlying problem and availability of system information. A suitable model-based approach can be employed by local sensors for sensor validation, whenever prior information regarding the system model is available. Without any prior information, the redundancy of a distributed architecture can be exploited to identify any inconsistency in the fusion pool. However, redundancy based approaches may fail in the case in which multiple sensors simultaneously provide inconsistent data. The CU method can be used to consistently fuse spurious data coming from multiple sources. Yet, the method is computationally expensive and results in inappropriately large conservative fused results.* *The fault-tolerant methods can also be jointly applied to improve the fusion performance in the presence of inconsistencies and solve complex fusion problems according to practical demands. Table 3 summarizes the characteristics of fusion approaches for inconsistent data sources*.

## 6. Conclusions and Future Directions

In this paper, we reviewed and analyzed the theories and approaches for multisensor data fusion in a distributed architecture. The reasons for the dependencies of local sensor estimates are discussed and various fusion algorithms for correlated data sources are summarized. Both classic results and recent developments in distributed multisensor data fusion with the assumption of unknown correlation are analyzed. Several fault-tolerant approaches for identification and removal/fusion of inconsistent sensor data are also reviewed. The appropriateness of the fusion technique depends on the underlying problem and the established assumptions of each method. Based on literature review, future directions are summarized here:

- The algorithms for fusion under unknown correlation in literature are mostly devised for the two-sensor case. A general fusion framework for more than two data sources under unknown correlation is still an open research question.
- A major limitation of the distributed fusion methods is that almost all the methods described are based on the traditional KF framework. Investigating these methods within a more powerful framework, such as particle filter, may be an interesting topic.
- While some research has been done on an explicit characterization of correlation for low-dimensional data sources, a general description and mathematical model for unknown correlation of multiple data sources is still an open question.
- Another interesting topic is the use of neural network for estimating the unknown correlation among multiple sensors in distributed architecture.
- Detection and removal of inconsistent and spurious sensor estimates in a distributed fusion architecture under unknown correlation is also an interesting problem.
- Examining the distributed fusion algorithms for network of nonlinear systems under unknown uncertainties may be an open and challenging research direction.
- Lack of a standard evaluation framework to assess the performance of distributed fusion algorithms is another issue. Most of the fusion algorithms are either tested on simulated data with arbitrary assumptions or applied to a specific real-world problem.

## Figures and Tables

**Table 1:** Fused result and average computation time of different ellipsoidal methods.

	CI	LE	IEA	EI
Fused Result	$x_{CI}={\left[\begin{array}{cc} 0.899 & 1.80 \end{array}\right]}^{T}$	$x_{LE}={\left[\begin{array}{cc} 0.82 & 1.60 \end{array}\right]}^{T}$	$x_{IEA}={\left[\begin{array}{cc} 1 & 2 \end{array}\right]}^{T}$	$x_{EI}={\left[\begin{array}{cc} 0.52 & 1.41 \end{array}\right]}^{T}$
	$P_{CI}=\left[\begin{array}{cc} 3.97 & 1.48\\ 1.48 & 3.23 \end{array}\right]$	$P_{LE}=\left[\begin{array}{cc} 3.33 & 0.98\\ 0.98 & 2.49 \end{array}\right]$	$P_{IEA}=\left[\begin{array}{cc} 4 & 1.8\\ 1.8 & 3.5 \end{array}\right]$	$P_{EI}=\left[\begin{array}{cc} 3.33 & 0.98\\ 0.98 & 2.49 \end{array}\right]$
	$\det\left(P_{CI}\right)=10.663$	$\det\left(P_{LE}\right)=7.365$	$\det\left(P_{IEA}\right)=10.76$	$\det\left(P_{EI}\right)=7.365$
Time (ms)	1.1668	0.1514	0.2879	0.2353

**Table 2:** Summary of various algorithms for fusion under unknown correlation.

Framework	Algorithms	Characteristics
**Data Decorrelation**	Double counting removal [27,28,76,77]	Tracking and explicitly removing the double counting Assumes a particular network topology Neither scalable nor practical solution for a large network of sensors
	Measurement reconstruction [25,26]	Decorrelating the sequence of measurements by reconstructing the measurements at fusion node Internal information like Kalman gain, association weights, and sensor model information etc. are required to reconstruct the measurements Inefficient and impractical for large distributed sensor networks
**Modeling Correlation** [29,30,31,32,84]		Approximate the unknown cross-covariance based on a function of correlation coefficient A closed form solution for scalar-valued and approximate solution for fusion of vector-valued two estimates Improved fusion performance by incorporating knowledge of cross-correlation Difficult to interpret cross-correlation for multiple estimates
**Ellipsoidal Methods**	Covariance Intersection Method [14,33,34,35]	Provides a consistent and minimum bound for two data sources Does not provide a tight overestimate for more than two data sources Computationally demanding
	Largest Ellipsoid Method [36]	Provides a less conservative estimate of fused covariance than CI Fused mean value is based on the independent assumption of KF
	Internal Ellipsoidal Approximation [37,38]	Approximate the fused covariance by an internal maximum ellipsoid Based on heuristics
	Ellipsoidal Intersection Method [39]	The fused mean and covariance is calculated based on mutual and exclusive information of the two data sources Less conservative than CI but may provide inconsistent fused results in some cases Limited to the fusion of two data sources

**Table 3:** Overview of the methodologies for inconsistent and spurious data sources.

Approaches	Characteristics
Model based approaches [121,122,125,132,135,137,139]	Identification and subsequent removal of inconsistent and abnormal data Uses residuals generated between the modeled outputs and actual sensor measurements to identify inconsistency Need prior information and limited to specific failure model(s)
Redundancy based approaches [40,41,43,47,141,143,144]	Uses consistency checking and majority voting to identify inconsistency among multiple sensor estimates of the same state Identification of corrupted sensor estimates without prior information May fail if inconsistent estimates are provided by multiple sensors simultaneously
Fusion based approaches [48,49]	Provides a consistent fused result as long as one estimate is consistent May results in inappropriately conservative fusion solution Computationally demanding
